# Immune‐independent acquired resistance to PD‐L1 antibody initiated by PD‐L1 upregulation via PI3K/AKT signaling can be reversed by anlotinib

**DOI:** 10.1002/cam4.6195

**Published:** 2023-06-23

**Authors:** Yuan Gao, Yingfang Feng, Shaochuan Liu, Yan Zhang, Jing Wang, Tingting Qin, Peng Chen, Kai Li

**Affiliations:** ^1^ Tianjin Medical University Cancer Institute and Hospital National Clinical Research Center for Cancer Tianjin China; ^2^ Key Laboratory of Cancer Prevention and Therapy Tianjin China; ^3^ Tianjin's Clinical Research Center for Cancer Tianjin China; ^4^ Department of Thoracic Oncology Tianjin Lung Cancer Center Tianjin Cancer Institute & Hospital Tianjin Medical University Tianjin China

**Keywords:** anlotinib, drug resistance, melanoma, PD‐L1, PI3K/AKT, VECs

## Abstract

Despite the benefit with cancer immunotherapies in clinical implication, immunotherapeutic resistance occurred in many patients and the mechanism remains unknown. Increasing evidence has revealed that cell‐intrinsic programmed cell death ligand 1 (PD‐L1) may play a non‐negotiable part in immunotherapeutic resistance. Our present study aimed to elucidate the immune‐independent acquired resistance mechanism to PD‐L1 antibody. We found elevated PD‐L1 expression induced by PD‐L1 antibodies in cancer cell and vascular endothelial cells (VECs) with substantially acquired resistance to PD‐L1 antibodies. Moreover, proliferation of resistant cells was accelerated and the apoptosis was reduced in the absence of immune compared with parental cells. Subsequently, we confirmed that the activation of the PI3K/AKT pathway is involved in the upregulation of PD‐L1 expression. Finally, we found that low dose of anlotinib downregulated PD‐L1 expression only in VECs via inhibiting the PI3K/AKT pathway; however, the same effect was not observed in cancer cells. To sum up, our findings revealed that upregulation of PD‐L1 via activation of the PI3K/AKT signal pathway may promote acquired resistance to PD‐L1 antibodies in an immune‐independent manner.

**Significance:**

These findings provide new mechanisms of immunotherapeutic resistance and effective evidence of anlotinib combined with immunotherapy.

## 1 INTRODUCTION

Programmed cell death ligand 1 (PD‐L1), an immunoinhibitory receptor, is ubiquitously expressed in multiple cancer types and is associated with clinicopathological characteristics and prognosis.^1^, ^2^, ^3^, ^4^ The current understanding of PD‐L1 mechanism is binding to PD‐1, counteracting T‐cell overactivation and mediating cancer immune evasion. Therefore, antibodies to PD‐L1 have considerably improved the survival in many malignancies over the past years.^5^, ^6^, ^7^ However, resistance to immunotherapy inevitably occurs in numerous cases. Although a series of studies have demonstrated that T‐cell exclusion, neoantigen loss, and activation of bypassed immune checkpoints were closely relevant with the acquired resistance of anti‐PD‐L1, the mechanisms are still not well understood. Qu et al. demonstrated NLRP3 inflammasome drives acquired resistance to anti‐PD‐1 antibodies in response to upregulation of PD‐L1 expression.^8^ Additionally, our prior study illustrated that increased expression of PD‐L1 in endothelial cells could prevent the infiltration of CD8^+^ effector T cells and promote the aggregation of Foxp3^+^ regulatory T cells, which lead to forming an immunosuppressive barrier and an immunosuppressive microenvironment.^9^

Recent studies discovered a potential “reverse signaling” effect of PD‐L1. For instance, tumor PD‐L1 is refractory to apoptosis,^10^ enhanced tumor cell glycolysis,^11^ maintaining its stemness,^12^ and promoting cell growth and autophagy.^13^, ^14^ Nevertheless, there is still a lot of controversy and obscurity regarding mechanism of the elevation of PD‐L1. Most studies focused on the TME, which could accelerate immune escape through the upregulated PD‐L1 in cancer cells induced by various pro‐inflammatory cytokines such as interferon gamma, TNFα, and IL‐6.^15^, ^16^, ^17^, ^18^ However, whether anti‐PD‐L1 antibody could directly induce the upregulation of PD‐L1 expression in an immune‐independent manner and the role of PD‐L1 intrinsic effect in the development of resistance to immune checkpoint inhibitors are unclear.

Anlotinib, a multitargeted tyrosine kinase inhibitor, has shown promising curative effects in extensive tumor treatment.^19^, ^20^, ^21^ Our previous study revealed that anlotinib could downregulate PD‐L1 in vascular endothelial cells (VECs) by inhibiting AKT phosphorylation to improve the immune microenvironment.^9^ Clinically, it produces synergistic therapeutic effects for radioimmunotherapy to achieve better antitumor potency in non‐small‐cell lung cancer.^22^ These evidences suggested that anlotinib may improve sensitivity to immune checkpoint inhibitors by regulating PD‐L1 expression.

In the current study, we demonstrated that the expression of hypoxia‐inducible factor (HIF)‐1α was upregulated in both tumor cells and VECs upon the induction of PD‐L1 antibodies. Furthermore, activation of the PI3K/AKT pathway could promote the upregulation of vascular endothelial growth factor A (VEGFA) and PD‐L1. Moreover, resistant cells exhibited accelerated proliferation and reduced apoptosis in vitro. Although low‐dose anlotinib decreased PD‐L1 in VECs, it had little effect on tumor cells. In summary, this work reveals new insights that the immune‐independent properties of PD‐L1 may play important roles in the development of resistance to immune checkpoint inhibitors. And our study also provides a promising way for low‐dose anlotinib to overcome resistance to PD‐L1 antibodies.

## 2 MATERIALS AND METHODS

### 2.1 Cell culture and generation of drug‐resistant cells

B16‐F10 cells were generously provided by Dr. Yuan from Tianjin Medical University Cancer Institute and Hospital. Human umbilical vein endothelial cells (HUVECs) were obtained from the Peking Union Medical College cell bank, and immortalized mouse brain endothelial cell lines (bEnd.3) were obtained from Nankai University. The B16‐F10, HUVEC, and bEnd.3 were all maintained in a complete medium with 90% DMEM and 10% fetal bovine serum at 37°C humidified incubator with 5% CO_2_ atmosphere. Anlotinib and TQB2450 (anti‐PD‐L1 antibody) were purchased from Nanjing Chia Tai Tianqing Company. Atezolizumab was bought from Roche. TQB2450‐resistant B16‐F10, HUVECs, and bEnd.3 cell lines, B16‐F10R, HUVECR, and bEnd.3R were established in our laboratory. Parental cells were seeded into 96‐well plates with 300–500 cells per well and added different gradients of TQB2450 (0, 0.1, 1, 10, 100, 500, and 1000 μg/mL). The cells were gradually expanded in culture from 96‐well plates to 24‐well plates, 6‐well plates, and finally to 6 cm dishes. These cells were further treated with stepwise increasing concentrations of TQB2450 over 3 months until the B16‐F10 cells survived in 300 μg/mL TQB2450 (5 folds IC50), and HUVECs and bEnd.3 cells survived in 100 μg/mL TQB2450 (5 folds IC50). Cell viability assay and stock cultures of these resistant cells were all maintained at the corresponding concentration of TQB2450 for future experiments. Similarly, we established atezolizumab‐resistant B16‐F10 cells using the above method.^23^

### 2.2 Cell viability assay and clonogenic assay

The sensitivity of B16‐F10, bEnd.3, and HUVEC parental cells or cells resistant to drugs was assessed using an MTT assay. These cells were planted into 96‐well plates with 1000 cells per well and treated with TQB2450 or anlotinib at various concentrations for 24–48 h. The mixture was added to the MTT reagent, followed by continuous culture for 4 h. The supernatants were removed and treated with 150 μL DMSO per well. Cell viability was assessed by the absorbance at 490 nm.

For the colony formation assay, 6‐well plates containing about 1500 cells each well were cultured with 2 mL complete medium in the presence or absence of TQB2450 for 12–24 h. Subsequently, fresh complete medium continuously treated for 10–14 days. Cell colonies were counted and photographed.

### 2.3 Flow cytometry analysis of apoptosis

Cell apoptosis was assessed by Annexin V‐FITC and PI staining kit (Yeasen Biotechnology Co., Ltd.). The cells were incubated with or without TQB2450 for 24–48 h. Subsequently, the cells were resuspended in binding buffer. Then, the corresponding reagents were added according to the instructions. The samples were evaluated using FACS Canto II (BD) within 1 h.

### 2.4 Quantitative real‐time PCR

Total RNA was isolated from cells using TRIzol (15596–018; Invitrogen) and synthesis of cDNA using the PrimeScript™ RT Master Mix from TaKaRa. RT‐qPCR reactions were performed using SYBR Premix Ex TaqTM II (TaKaRa). The expression of the target genes was calculated using the 2^−ΔΔCt^ method. The sequences of the primers were listed in the supporting Table S1.

### 2.5 Western blotting

Protein lysates were obtained in RIPA buffer containing protease and phosphatase inhibitors (Thermo Fisher Scientific). Cells were lysed, and cell membrane, cytoplasmic, and nuclear proteins were separated and extracted using a Minute (TM) Plasma Membrane Protein Isolation and Cell Fractionation Kit and Minute (TM) Denaturing Protein Solubilization Reagent (Invent Biotechnologies). Protein concentrations were quantified using the BCA assay kit (Thermo Fisher Scientific). The proteins were separated on SDS‐PAGE gels, then transferred to PVDF membranes (Roche Molecular Biochemicals), and probed with indicated antibodies against human PD‐L1 (ab205921; Abcam, 1:1000), mouse PD‐L1(ab213480; Abcam, 1:1000); mouse BCL‐2 (#3498; Cell Signaling Technology, 1:1000), human BCL‐2 (#4223; Cell Signaling Technology, 1:1000); AKT (#4685; CST, 1:1000); p‐AKT (Ser473) (#4060; CST, 1:1000); PI3K (#4255; CST, 1:1000); mouse p‐PI3K (Tyr458/Tyr199) (#4228; CST, 1:1000), human p‐PI3K (PI3K p85 + PI3 Kinase p55) (ab278545; Abcam, 1:1000); VEGFA (ab46154; Abcam, 1:1000); human BAX (#5023; CST, 1:1000), mouse BAX (#14796; CST, 1:1000); human HIF‐1α (20960‐1‐AP; Proteintech, 1:1000), mouse HIF‐1α (#48085; CST, 1:1000); Na, K‐ATPase α (#3010; CST, 1:1000); Histone H3 (#4499; CST, 1:2000); GAPDH (#5174; CST, 1:1000). The second day, the membranes were incubated with rabbit or mouse secondary antibodies and visualized using the chemiluminescence system (Millipore and Epizyme).

### 2.6 Transfection

Stable B16‐F10 and bEnd.3 cells silenced and overexpressed for CD274 were generated using lentiviral constructs expressing shCD274 (GGCGTTTACTGCTGCATAA), OE‐CD274 and negative control (GeneChem Co. Ltd.), and maintenance in puromycin (Selleck). The transfection efficiency was verified by using western blot assay.

### 2.7 Statistical analysis

All experiment trials were repeated three times independently. All assay data are presented as mean ± standard deviation, and the differences between the two groups were evaluated using unpaired Student's *t*‐test. Statistical analyses were performed using GraphPad Prism 8.0 software (GraphPad Software).

## 3 RESULTS

### 3.1 Elevated PD‐L1 was found in the cells with acquired resistance to PD‐L1 antibodies

**FIGURE 1:**
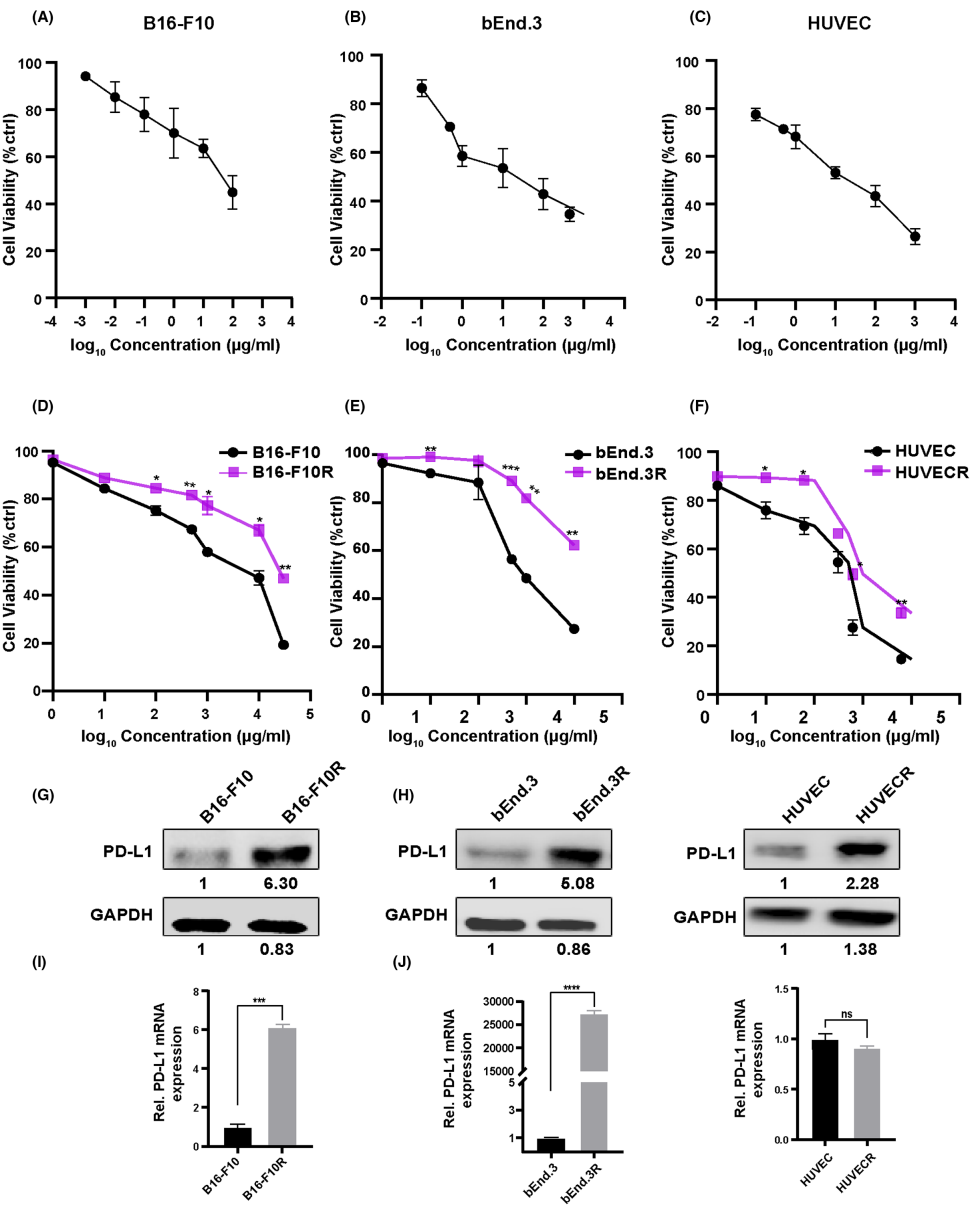
Development of acquired resistance to PD‐L1 antibody is associated with elevated PD‐L1 expression. (A–C) Proliferation of B16‐F10, bEnd.3, and HUVECs were detected by MTT assay after treatment with TQB2450 at different concentrations. (D–F) Effect of different gradients of TQB2450 on cell proliferation of B16‐F10/B16‐F10R, bEnd.3/ bEnd.3R, and HUVEC/ HUVECR cells. (G–J) Expression of PD‐L1 in B16‐F10/B16‐F10R, bEnd.3/ bEnd.3R, and HUVEC/ HUVECR cells were detected by western blot (upper panel) and qRT‐PCR (lower panel).

To investigate the detailed impact of PD‐L1 antibody on cell viability in vitro, different concentrations of TQB2450 were applied on B16‐F10 cells. The proliferation of B16‐F10 cells was inhibited by PD‐L1 antibody in a dose‐dependent manner (Figure 1A). Similarly, PD‐L1 antibody also inhibited cell proliferation of bEnd.3 and HUVEC cells in a dosage‐dependent way (Figure 1B,C). However, with prolonged treating time, B16‐F10, bEnd.3, and HUVECs cells all regrew following the inhibition of proliferation (Supplementary Figure S1A–C). To elucidate the immune‐independent mechanism of resistance to PD‐L1 blockade immunotherapy. B16‐F10, bEnd.3, and HUVEC cells were exposed to PD‐L1 antibody (TQB2450) at gradually increasing concentrations for more than 3 months to establish PD‐L1 antibody‐resistant cells (B16‐F10R, bEnd.3R, and HUVECR, respectively). Cell viability assay demonstrated that B16‐F10R, bEnd.3R, and HUVECR cells were completely insensitive to the PD‐L1 antibody compared with their respective parental cells (Figure 1D–F). Moreover, western blotting assays showed that the expression of PD‐L1 conspicuously increased in resistant cells in comparison with their parental cells (Figure 1G,H). Similarly, we also established atezolizumab‐resistant B16‐F10 cells and further confirmed that PD‐L1 protein was markedly increased compared with that in the parental cells (Supplemental Figure S2). Several recent reports have uncovered that redistribution of PD‐L1^24^ or increased incorporation of PD‐L1 into the nucleus^25^ plays an important role in promoting tumorigenesis and increasing resistance to chemotherapy and immunotherapy.^26^ Thus, we extracted proteins from the membrane, cytoplasm and the nucleus of the cells and measured the expression of PD‐L1 in these fractions. The results showed that, compared with the respective parental cells in B16‐F10R and bEND.3R cells, the expression of PD‐L1 in the membrane, cytoplasm and nuclear were all increased. This is consistent with our findings that both the mRNA and protein expression of PD‐L1 were significantly increased in B16‐F10R and bEND.3R cells. (Figure 1G–J, Supplemental Figure S3). The expression of PD‐L1 in membrane and cytoplasm was decreased and, however, increased in the nuclear fraction in HUVECR cells in correspondence with our results that total protein expression of PD‐L1 was increased, while mRNA expression levels were not evidently altered to the control group (Figure 1H,J, Supplemental Figure S3). These data clearly demonstrated that cells could develop immune‐independent acquired resistance to PD‐L1 antibodies, following PD‐L1 upregulation.

### 3.2 PD‐L1 promotes proliferation in an immune‐independent manner in vitro

**FIGURE 2:**
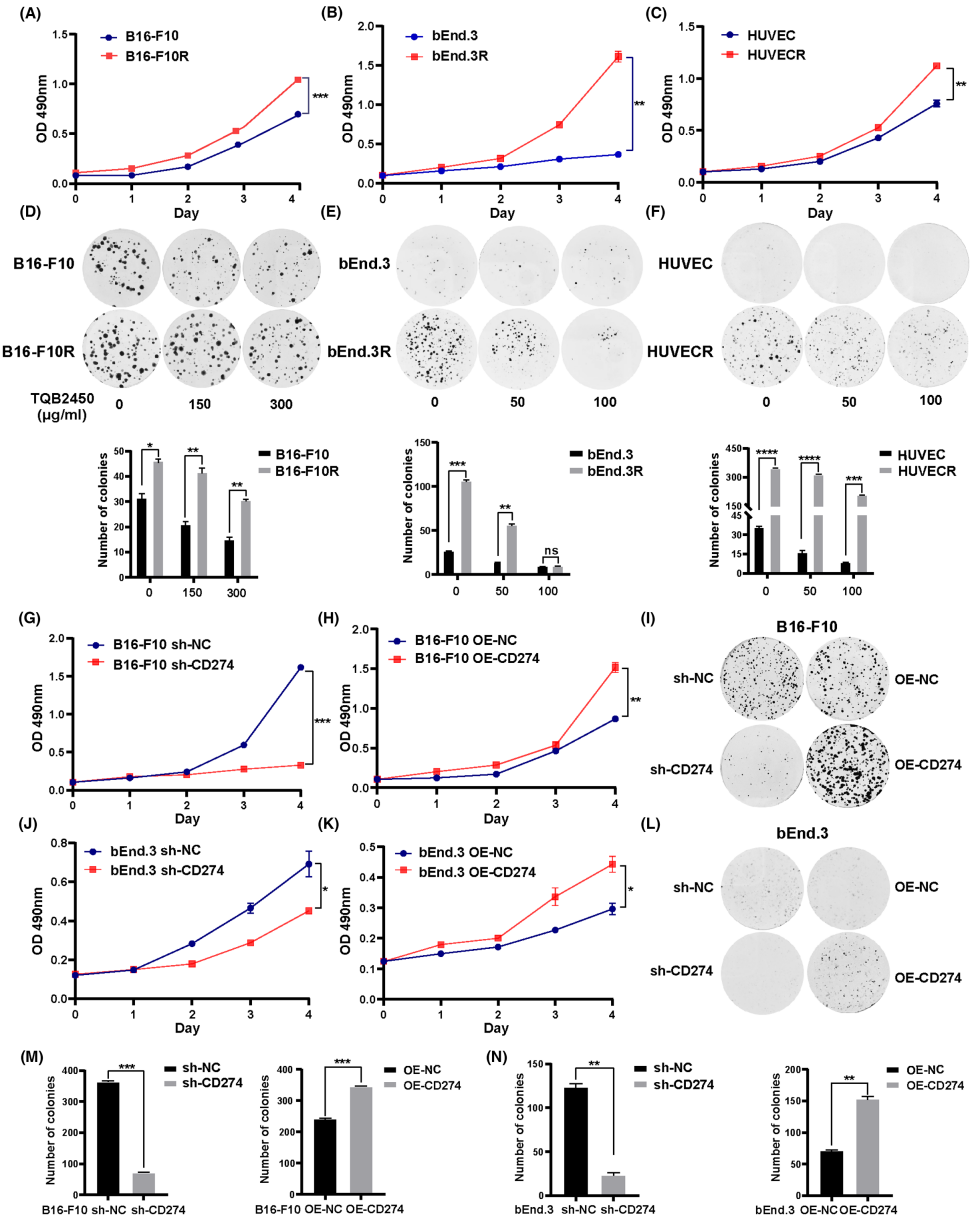
PD‐L1 promotes immune‐independent proliferation of the tumor cells and VECs in vitro. (A–C) Proliferation in vitro of B16‐F10R, bEnd.3R, and HUVECR cells determined by MTT assay versus their parental cells. (D–F) The colony formation ability in different gradients of TQB2450 treatment on B16‐F10/B16‐F10R, bEnd.3/bEnd.3R, and HUVEC/HUVECR cells was measured. (G–I, M) MTT and colony formation assays were used to detect growth and proliferation of B16‐F10 cells with stable overexpression and knockdown of CD274. (J–L, N) In vitro proliferation of bEnd.3 cells on CD274‐KD and CD274‐OE validated by MTT and colony formation assays compared with controls, respectively. Data are presented as mean ± SD. *p < 0.05, **p < 0.01, ***p < 0.001, ****p < 0.0001, Student's *t*‐test.

To further confirm the increased PD‐L1 expression in B16‐F10R, bEnd.3R, and HUVECR with a complete absence of an adaptive immune response has an impact on cell proliferation. B16‐F10R cells proliferated significantly faster than the control B16‐F10 cells in vitro (Figure 2A). Our assay revealed that the number of colonies increased significantly after the occurrence of resistance to PD‐L1 antibody (Figure 2D). This conforms with the previous research results. Tumor‐inherent PD‐L1 could regulate the proliferation of ID8agg and B16‐F10 in the absence of tumor‐specific immunity.^14^, ^27^ Consistent with B16‐F10R cells, bEnd.3R and HUVECR cells, all highly expressed PD‐L1, also showed stronger proliferation viability and clonogenic ability in the absence of tumor immunity response. (Figure 2B,C,E,F). We used lentivirus transfection to construct B16‐F10 and bEnd.3 cell lines with stable overexpression or knockdown of PD‐L1, and the efficiency was verified by western blotting (Supplementary Figure S6). MTT and cell clone formation assays were used to evaluate the cell proliferation of B16‐F10 and bEnd.3. The results demonstrated that CD274‐KD impaired and CD274‐OE promoted proliferation compared with the respective control cells (Figure 2G–N). Taken together, these discoveries suggested the role of PD‐L1 in promoting the proliferation of resistant cells.

### 3.3 PD‐L1 induces immune‐independent anti‐apoptosis in vitro

**FIGURE 3:**
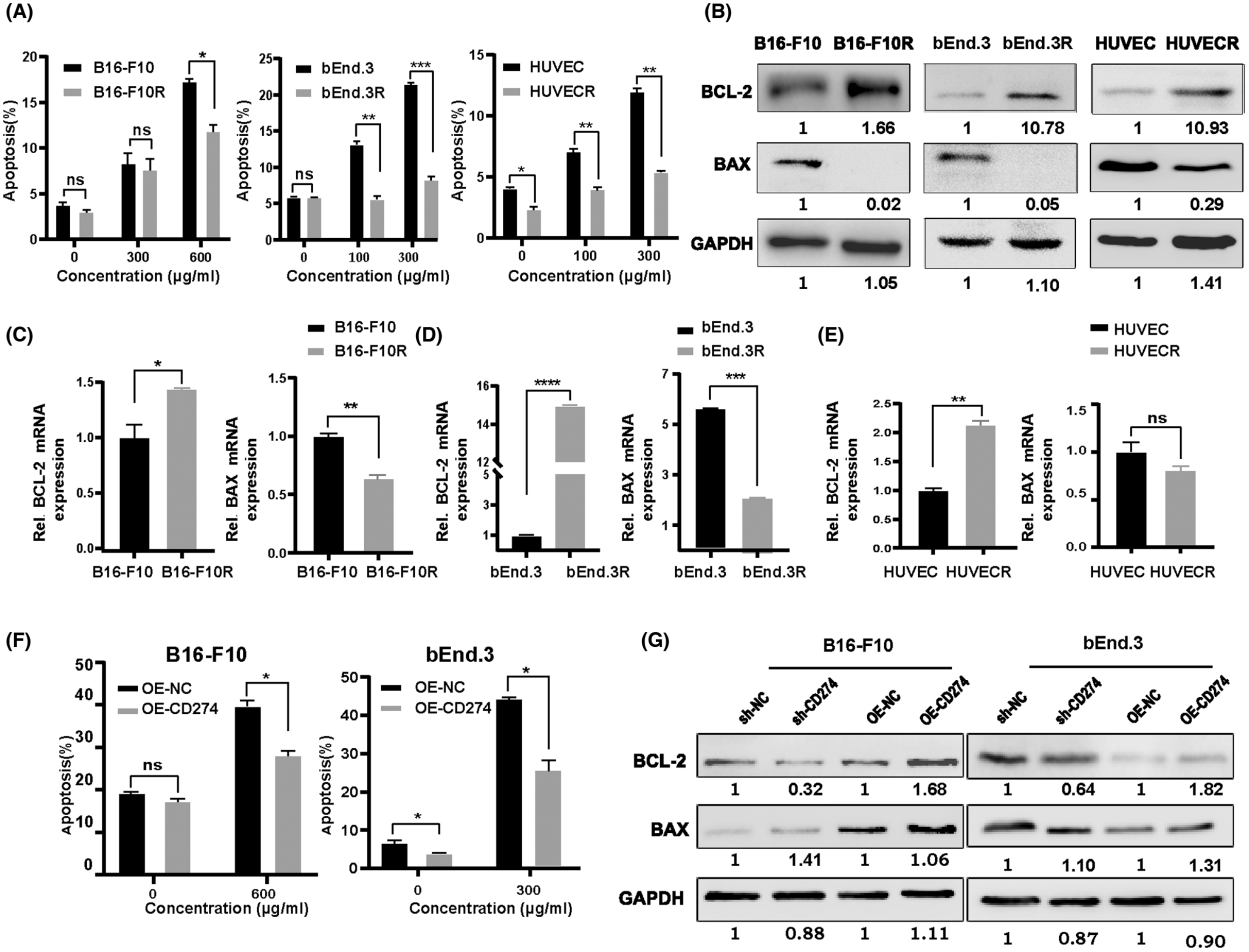
PD‐L1 inhibits immune‐independent apoptosis of tumor cells and VECs in vitro. (A) Apoptosis of B16‐F10/B16‐F10R, bEnd.3/ bEnd.3R, and HUVEC/ HUVECR cells was detected by flow cytometry after treatment with TQB2450. (B–E) The protein and mRNA expressions of BCL‐2 and BAX were detected in PD‐L1 antibody‐resistant cells and parental cells. (F) Flow cytometry was used to assess apoptosis of B16‐F10 and bEnd.3 cells with stable overexpression CD274 treated with TQB2450. (G) The abundance of BCL‐2 and BAX was measured in CD274‐KD and CD274‐OE of B16‐F10 and bEnd.3 cells. Data are presented as mean ± SD. *p < 0.05, **p < 0.01, ***p < 0.001, ****p < 0.0001, Student's *t*‐test.

To further verify whether PD‐L1 overexpression in B16‐F10R cells affects the apoptosis in the absence of immune cells in vitro, flow cytometry was performed. After incubation of B16‐F10R cells with PD‐L1 antibody for 48 h, the apoptotic cells remarkably decreased compared with the control (Figure 3A, Supplemental Figure S4). Our assays also assessed apoptosis‐associated gene expression. The mRNA and protein expression of BCL‐2 were distinctly upregulated and BAX downregulated in B16‐F10R cells when compared to parental cells (Figure 3B,C). In accordance with previous reports that PD‐L1 in tumor cells is an antiapoptotic receptor with inhibition of antitumor immune responses,^10^ we further explored whether PD‐L1 overexpression in nontumoral cells would also confer resistance to apoptosis. The apoptosis rates of bEnd.3, bEnd.3R, and HUVEC and HUVECR cells were determined after incubation of the PD‐L1 antibody for 24 h. The results showed that the apoptosis of bEnd.3 and HUVEC increased in a dose‐dependent manner, while bEnd.3R and HUVECR exhibited lower pro‐apoptotic activity at the same concentration (Figure 3A, Supplemental Figure S4). The expression levels of BCL‐2 and BAX mRNA and protein in bEnd.3R and HUVECR are consistent with the results in cancer cells (Figure 3B,D,E). The above findings revealed that cells with elevated expression of PD‐L1 have immune‐independent antiapoptotic activity. To confirm that PD‐L1 overexpression was associated with resistance to apoptosis, we used CD274‐OE B16‐F10 and bEnd.3 cells to verify the above findings. In accordance with the aforementioned results, diminished apoptosis and increased BCL‐2 were observed in the OE‐CD274 group compared with those in control (Figure 3F,G, Supplemental Figure S5). Taken together, PD‐L1‐mediated antiapoptosis was independent of immunoreaction and occurred in tumor cells as well as in VECs.

### 3.4 PD‐L1 upregulation is associated with the activation of PI3K/AKT pathway

**FIGURE 4:**
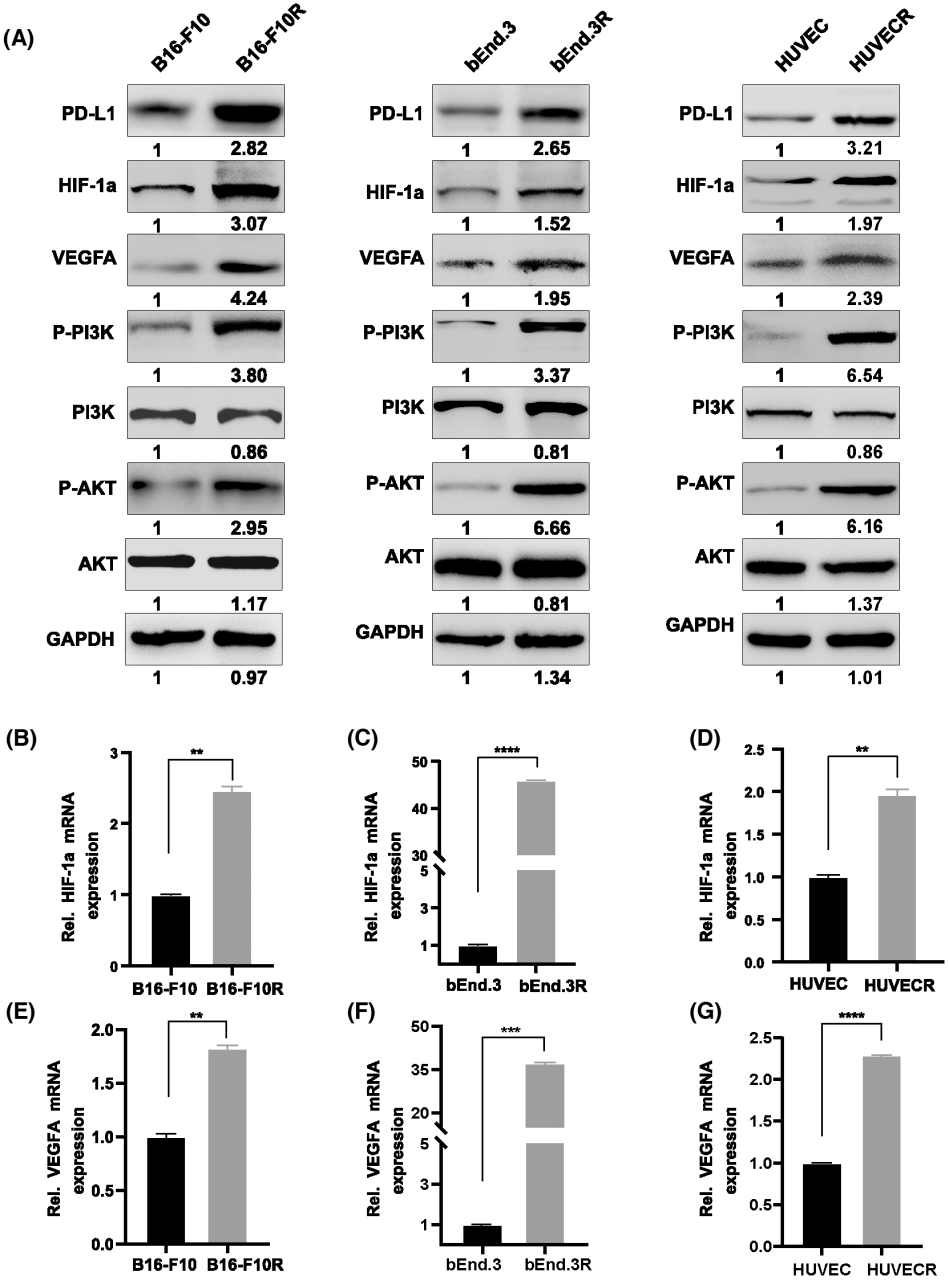
PD‐L1 mediates immune‐independent resistance to PD‐L1 antibody involved in the PI3K/AKT pathway. (A) Western blot analysis of p‐PI3K, p‐AKT, HIF‐1α, VEGFA, and PD‐L1 in B16‐F10/B16‐F10R, bEnd.3/ bEnd.3R, and HUVEC/ HUVECR cells. (B–G) The results of qPCR revealed increased mRNA expression of HIF‐1α and VEGFA in resistant cells. Data are presented as the mean ± SD. *p < 0.05, **p < 0.01, ***p < 0.001, ****p < 0.0001, Student's *t*‐test.

**FIGURE 5:**
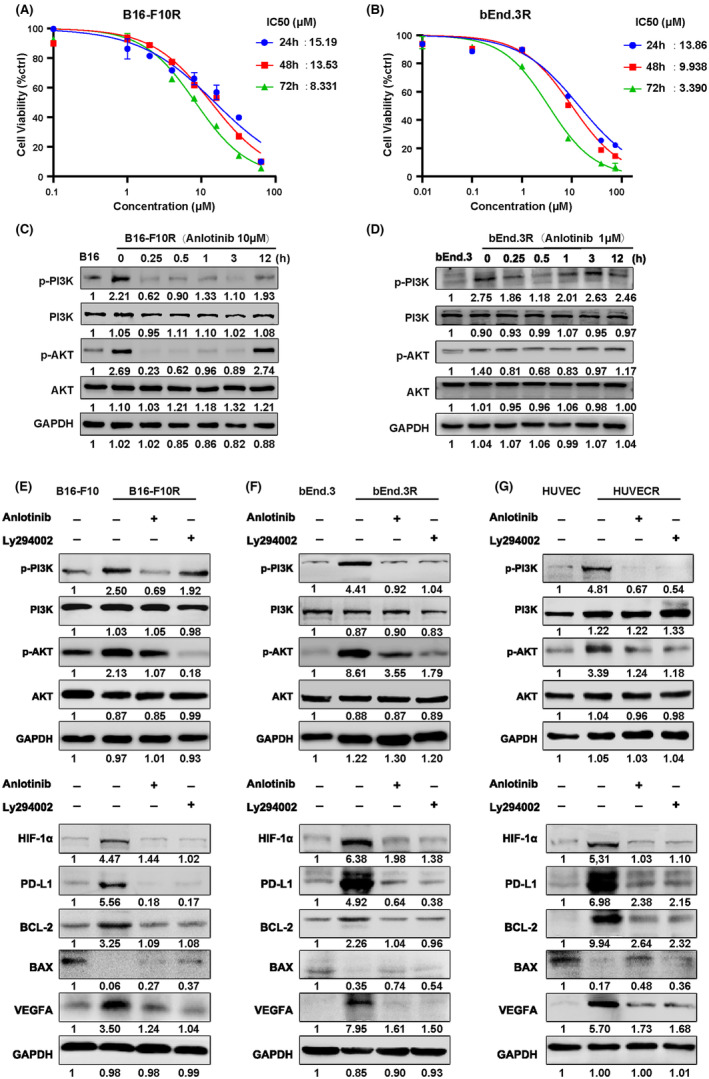
Anlotinib downregulates endothelial and tumorous PD‐L1 expression via the PI3K/AKT pathway. (A,B) PD‐L1 antibody‐resistant B16‐F10 and bEnd.3 cells were exposed to various concentrations of anlotinib for 24, 48, 72 h, respectively. Then, the IC_50_ values of anlotinib in the indicated groups were measured by MTT assay. (C,D) Western blot analysis showed p‐PI3K and p‐AKT levels in with or without anlotinib (10 μM)‐treated B16‐F10R and anlotinib (1 μM)‐treated bEnd.3R at the indicated time points. (E–G) Western blot analysis confirmed PD‐L1, HIF‐1α, VEGFA and related apoptosis protein expression levels in B16‐F10R, bEnd.3R, and HUVECR cells treated by anlotinib (B16‐F10R with 10 μM, and bEnd.3R and HUVECR with 1 μM), Ly294002 (AKT inhibitor, 10 μM) at 24 h and 48 h. Western blot analysis showed p‐PI3K and p‐AKT levels treated with anlotinib (10 μM) in B16‐F10R at 0.25 h, anlotinib (1 μM) in bEnd.3R and HUVECR cells at 0.5 h. Data are presented as mean ± SD. *p < 0.05, **p < 0.01, ***p < 0.001, ****p < 0.0001, Student's *t*‐test.

A previous study indicated that PD‐L1 expression relies on the activation of the PI3K/AKT pathway.^9^ Western blotting protein analysis revealed that the elevated PD‐L1 was accompanied by the phosphorylation of the PI3K/AKT pathway in resistant cells (Figure 4A). Additionally, PD‐L1 protein level was decreased by an AKT inhibitor (LY294002; Figure 5E–G), suggesting that the PI3K/AKT signaling mediates PD‐L1 expression. Previous studies have confirmed that HIF‐1α directly regulated the transcription of PD‐L1 and VEGFA by binding the hypoxic promoter region (HER).^28^, ^29^ Consistently, both qRT‐PCR and western blotting assays proved that the expression of HIF‐1α was positively correlated with PD‐L1, accompanied by the simultaneous change of VEGFA (Figure 4A–G). These results suggested that the upregulation of PD‐L1 in resistant cells occurred through activation of the PI3K/AKT pathway.

### 3.5 Anlotinib inhibits PD‐L1 upregulation via PI3K/AKT Pathway

The IC_50_ of anlotinib in resistant cells was determined by MTT method. The defined dosage of anlotinib had little effect on cell proliferation, but downregulated PD‐L1 expression, was applied to the following experiments (Figure 5A,B). Anlotinib suppressed the phosphorylation of the PI3K/AKT pathway in time‐dependent manner (Figure 5C,D). Western blotting showed that anlotinib not only suppressed the phosphorylation of the PI3K/AKT signaling pathway but also downregulated PD‐L1 (Figure 5E–G). To validate regulatory mechanism of PD‐L1, the addition of AKT inhibitor (LY294002) could also inhibit PD‐L1 expression (Figure 5E–G). Thus, our results demonstrated that anlotinib could downregulate PD‐L1 expression in resistant cells by inhibiting the activation of the PI3K/AKT pathway. Interestingly, anlotinib could downregulate PD‐L1 in bEnd.3R and HUVECR cells but not in B16‐F10R cells at a low concentration of 1 μM (Supplementary Figure S7B), and a higher concentration of 10 μM was needed for substantial downregulation of PD‐L1 in B16‐F10R cells (Figure 5E, Supplementary Figure S7A).

## 4 DISCUSSION

Our current study demonstrated that cells may develop immune‐independent acquired resistance of long‐term PD‐L1 antibody at a specific concentration, which was associated with the upregulation of PD‐L1. Additionally, we showed that the activation of the PI3K/AKT pathway was involved in the PD‐L1 expression of resistant cells. Finally, we found that low concentration of anlotinib could decrease the expression of PD‐L1 only in VECs via inhibiting the PI3K/AKT pathway; however, the same effect was not observed in cancer cells (Supplementary Figure S8). These findings provide new mechanisms of immunotherapeutic resistance and evidence for the combination anlotinib with immunotherapy.

Although the application of immune checkpoint inhibitors improved the survival of many types of malignant tumors, a growing number of patients have developed immunotherapeutic resistance. Investigators have shown that the mechanisms of immunotherapy resistance mainly include two aspects: tumor and its microenvironment, such as tumor mutational load, oncogenic signaling pathways, hypoxia, and metabolism disorder of T cells,^30^ wherein PD‐L1 in tumor cells plays a vital part. Moreover, some attention has been paid to the direct effects of PD‐L1 antibody to block PD‐L1 on tumor cells independent of immunity. A previous study showed that atezolizumab suppressed proliferation and induced mitochondria‐related apoptosis in osteosarcoma cells in an immune‐independent manner.^31^ Similar results were found in our experiments, where cell proliferation was inhibited in a dosage‐dependent manner by PD‐L1 antibody (TQB2450). Then, cell resistance to PD‐L1 antibodies (TQB2450 and atezolizumab) was induced in vitro in a low‐dose‐escalation manner, and the rejection rate of resistant cells was significantly reduced compared with parental cells. This demonstrated that immune‐independent acquired resistance to PD‐L1 antibody induced by itself may also be involved in immunotherapy resistance.

PD‐L1, also well known as surface antigen cluster of differentiation 274 (CD274), is an immunosuppressive protein. The IgV of the ectodomain of PD‐L1 has a complementarity‐determining region that enables it to bind to PD‐1 in a 1:1 manner and induces a conformational change in PD‐1, thereby attenuating T‐cell activity, proliferation, and other effector functions.^32^ The latest studies have suggested that PD‐L1 has a T‐cell‐independent function. For example, PD‐L1 participates in regulating tumor cell metabolism, proliferation, and metastasis.^11^, ^13^, ^14^ Several reports have suggested that redistribution of PD‐L1^24^ or increased incorporation of PD‐L1 into the nucleus^25^ plays an important role in promoting tumorigenesis and increasing resistance to chemotherapy and immunotherapy.^26^ Our results showed that, in B16‐F10R and bEND.3R cells, the expression of PD‐L1 in the membrane, cytoplasm, and nuclear were all increased consistent with our findings that both the mRNA and protein expression of PD‐L1 were significantly increased. The expression of PD‐L1 in membrane and cytoplasm was decreased and, however, increased in the nuclear fraction in HUVECR cells in correspondence with our results that total protein expression of PD‐L1 was increased, while mRNA expression levels were not evidently altered to the control group. These results indicated that PD‐L1 may be redistributed in HUVECR cells, rather than the parallel change in transcript and protein levels.

However, whether PD‐L1 on tumor cells also has potential intrinsic effects to mediate immunotherapy resistance remains uncertain. Therefore, cell‐intrinsic PD‐L1 signaling needs to make further efforts. In this regard, our study revealed that cell proliferation viability and clone‐forming ability were significantly enhanced in resistant cells. We further demonstrated that resistant cells and PD‐L1 OE cells exhibited resistance to apoptosis in both tumor cells and VECs, similar to previous studies wherein PD‐L1 was an antiapoptotic receptor on cancer cells.^10^ Therefore, our results clearly show that cell‐intrinsic PD‐L1 has immune‐independent properties that may mediate resistance to immunotherapy.

Multiple oncogenic signaling pathways, including PI3K/AKT, are well known to be involved in the regulation of PD‐L1.^33^, ^34^, ^35^ In our finding, we also found that upregulation of PD‐L1 in resistant cells was accompanied by the phosphorylation of PI3K/AKT pathway, and the protein expression of PD‐L1 downregulated after treating with AKT inhibitor (Ly294002). This result suggested that the PI3K/AKT signaling pathway regulates PD‐L1 expression and may take part in PD‐L1‐mediated immune‐independent resistance. Additionally, HIF is an important regulatory subunit for its adaptation to hypoxic environments, which is involved in tumor formation, angiogenesis, and metastasis.^36^ Previous studies have indicated that activated PI3K/AKT signaling could upregulate HIF‐1α transcription and translation regardless of oxygen levels.^37^, ^38^ HIF‐1α could directly regulate the expression of PD‐L1 and VEGFA by binding to the hypoxic promoter region of the promoter.^28^, ^39^ In the current study, phosphorylation of PI3K/AKT signaling in resistant cells increased the expression of HIF‐1α along with high VEGFA and other factors participating in neovascularization, thereby further upregulating PD‐L1 expression (Supplementary Figure S8). We speculated that antiangiogenic drugs downregulate the expression of cellular PD‐L1. Studies have previously shown that bevacizumab could activate the phosphorylating of AKT pathway and ultimately leading to the upregulation of PD‐L1 expression, though it successfully inhibits VEGF signaling pathways.^40^, ^41^, ^42^ However, previous study indicated that anlotinib downregulated PD‐L1 in tumor cells and VECs via inhibiting the AKT pathway.^9^, ^43^ Our present results also showed that 1 μM anlotinib exerted the above function on resistant bEnd.3 cells by inhibiting PI3K/AKT pathway activation, while the same dose had less effect on tumor cells. Our results provide a strategy of abolishing the “immune barrier” of PD‐L1 on VECs and further maintaining PD‐L1 expression on tumor cells to be sensitive to PD‐L1 antibody to reinforce the immunotherapeutic efficacy.^9^ It also suggested a potential mechanism for the superiority of multitargeted antivascular drugs over single‐target inhibitors.

This study had some limitations. First, it is unclear whether anlotinib inhibits AKT only through its inhibition of VEGF, TGFβ, and FGF signaling or through other direct actions. Second, additional work is required to elucidate whether other molecules or modulation processes are involved. Last but not least, in vivo research using animal models is necessary to confirm our hypothesis, and our next study will carry out relevant in vivo experiments to further investigate the immune‐independent mechanism of acquired resistance of PD‐L1.

## 5 CONCLUSIONS

In summary, our study revealed that both tumor cell and vascular endothelium could develop resistance to PD‐L1 antibody with upregulation of PD‐L1 expression under long‐term induction of antibodies. It also illustrated the mechanism of PD‐L1 in the development of immune‐independent resistance and further explored the downregulation of anlotinib on PD‐L1 expression in tumors and VECs. As far as we know, this is the first study to confirm the elevation of PD‐L1 in tumor and vascular endothelial cells induced by the PD‐L1 antibody and reversed by anlotinib. This evidence may provide new references to abrogate immunotherapy resistance in the clinical setting.

## AUTHOR CONTRIBUTIONS

**Yuan Gao:** Conceptualization (equal); software (equal); validation (equal); writing – original draft (equal). **Yingfang Feng:** Formal analysis (equal); methodology (equal); validation (equal). **Shaochuan Liu:** Data curation (equal); formal analysis (equal); methodology (equal). **Yan Zhang:** Methodology (equal); validation (equal). **Jing Wang:** Funding acquisition (equal); project administration (equal). **Tingting Qin:** Funding acquisition (equal); resources (equal); supervision (equal). **Peng Chen:** Conceptualization (equal); resources (equal); supervision (equal); writing – review and editing (equal). **Kai Li:** Conceptualization (equal); funding acquisition (equal); project administration (equal); resources (equal); writing – review and editing (equal).

## FUNDING INFORMATION

This research was partially supported by grants from Tianjin Municipality Science and Technology Commission Projects (12ZCDZSY15600); CSCO (Chinese Society of Clinical Oncology) Special Foundation for Tumor Antiangiogenesis Therapy (Y‐X2011‐001); National Nature Science Foundation of China (Grant No. 82272686) and Nature Science Foundation of Tianjin (Grant No. 21JCYBJC01000); CSCO (Chinese Society of Clinical Oncology) Special Foundation for Tumor Antiangiogenesis Therapy (Y‐S2014‐011); Tianjin Key Medical Discipline (Specialty) Construction Project (TJYXZDXK‐010A).

## CONFLICT OF INTEREST STATEMENT

The authors declare no potential conflict of interest.

## Supporting information


Figure S1‐S8

Table S1

## Data Availability

The complete datasets of our current study are available from the corresponding author on reasonable request.
