# Supplementary material for: Immune‐independent acquired resistance to PD‐L1 antibody initiated by PD‐L1 upregulation via PI3K/AKT signaling can be reversed by anlotinib
Source: Cancer Med. 2023 Jun 23;12(14):15337–49. doi: 10.1002/cam4.6195 (PMC10417303; doi:10.1002/cam4.6195)

1. Supplementary figures

Supplementary Figure 1. Proliferation trend of B16-F10, bEnd.3 and HUVEC cells were detected by MTT assay after the treatment of TQB2450 with different concentrations and extension of time.

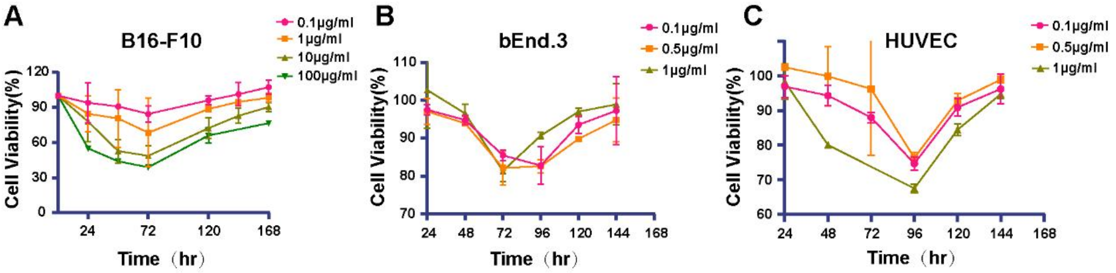

Supplementary Figure 2. Western blot analysis showed PD-L1 expression in B16-F10/B16-F10R after induced by Atezolizumab.

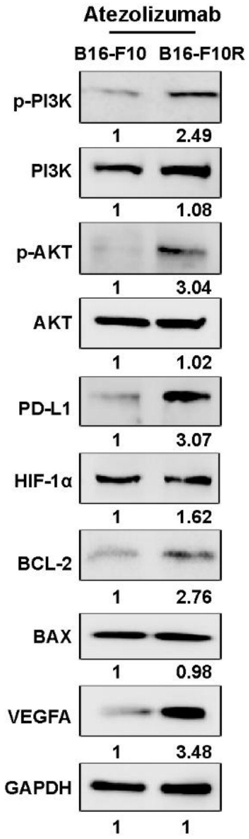

Supplementary Figure 3. Western blot analysis showed the distribution of PD-L1 in the membrane, cytoplasm and nuclear compared with the respective parental cells in B16-F10R, bEnd.3R and HUVECR cells.

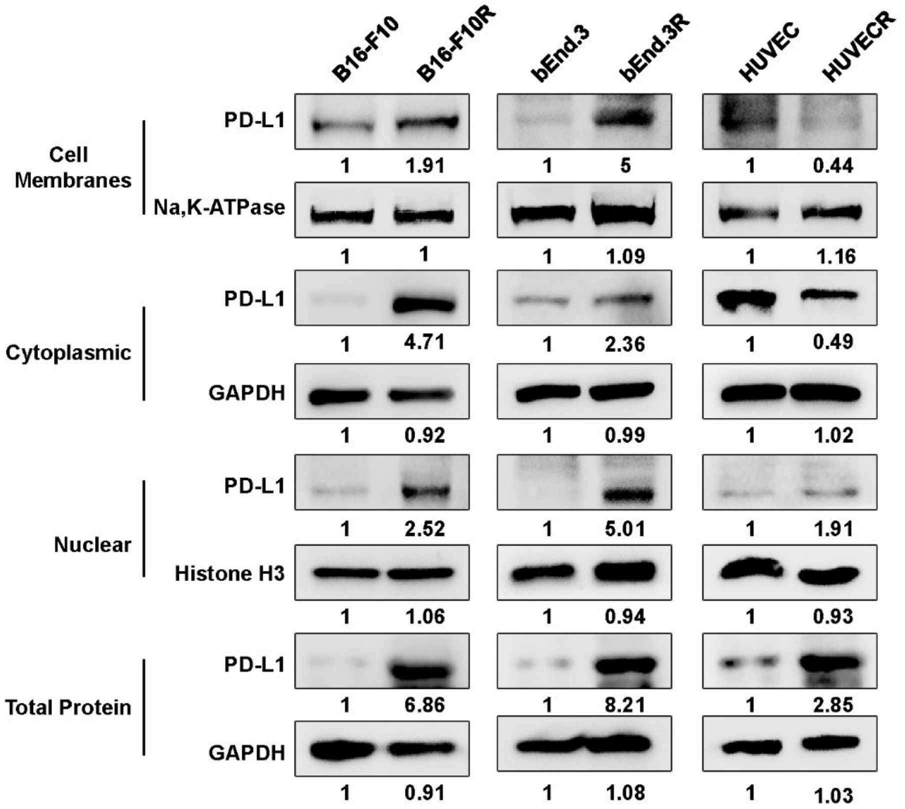

Supplementary Figure 4. Apoptosis of B16-F10/B16-F10R, bEnd.3/ bEnd.3R, and HUVEC/HUVECR cells was detected by flow cytometry after treatment with TQB2450.

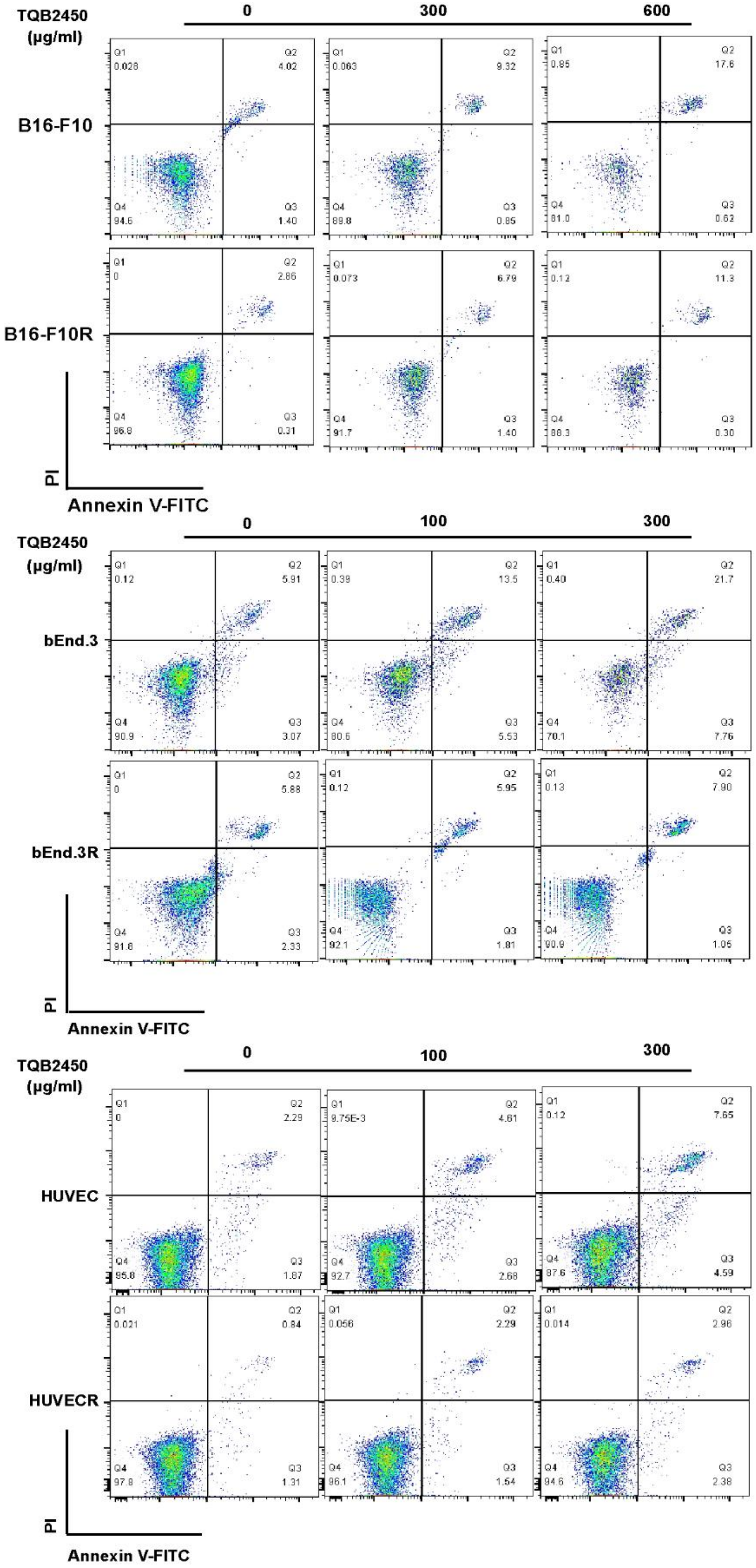
TQB2450
(μg/ml)
0
300
600
B16-F10
B16-F10R
PI
Annexin V-FITC
TQB2450
(μg/ml)
0
100
300
bEnd.3
bEnd.3R
PI
Annexin V-FITC
TQB2450
(μg/ml)
0
100
300
HUVEC
HUVECR
PI
Annexin V-FITC

Supplementary Figure 5. Flow cytometry was used to assess apoptosis of B16-F10 and bEnd.3 cells with stable overexpression CD274 treated with TQB2450.

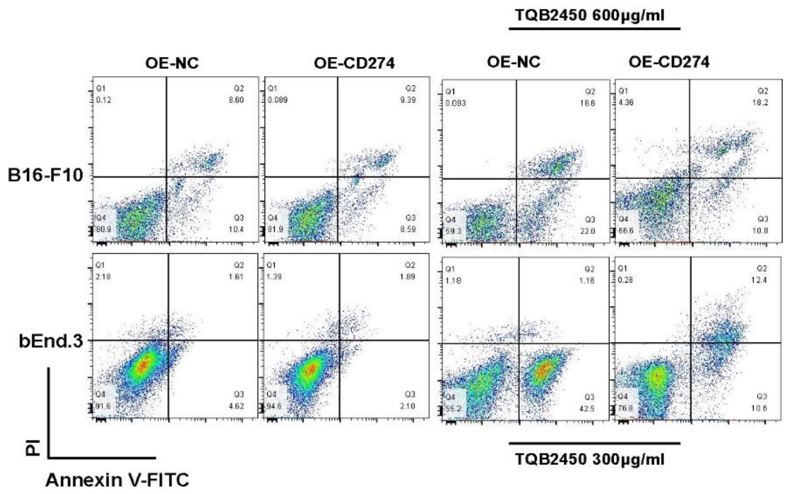

Supplementary Figure 6. Expression of PD-L1 was detected by western blot after lentivirus transfection to construct B16-F10 and bEnd.3 cell lines with stable overexpression or knockdown of CD274.

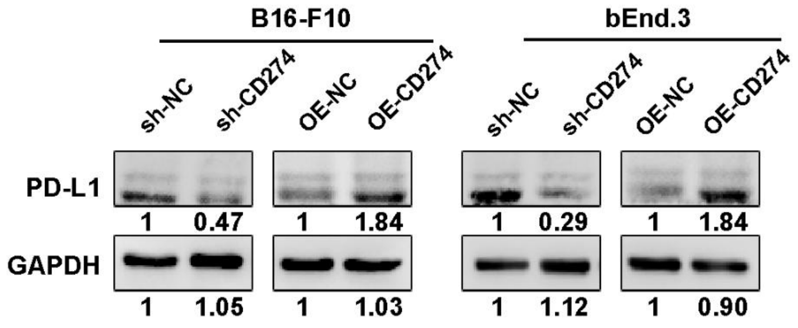

Supplementary Figure 7. A. Western blot analysis showed PD-L1 expression in B16-F10R after the treatment of Anlotinib with different concentrations. B. Western

blot differential expression confirmed PD-L1 protein expression levels in B16-F10R, bEnd.3R and HUVECR treated by Anlotinib 1 μM.

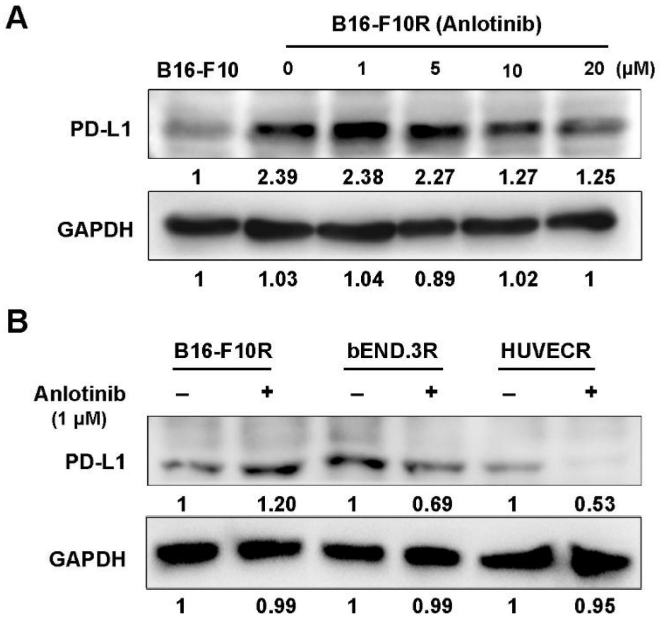

Supplementary Figure 8. A schematic representation of the mechanism by which PI3K/AKT pathway activation modulates PD-L1 antibodies resistance which decreases apoptosis and increases proliferation by elevating PD-L1 protein expression.

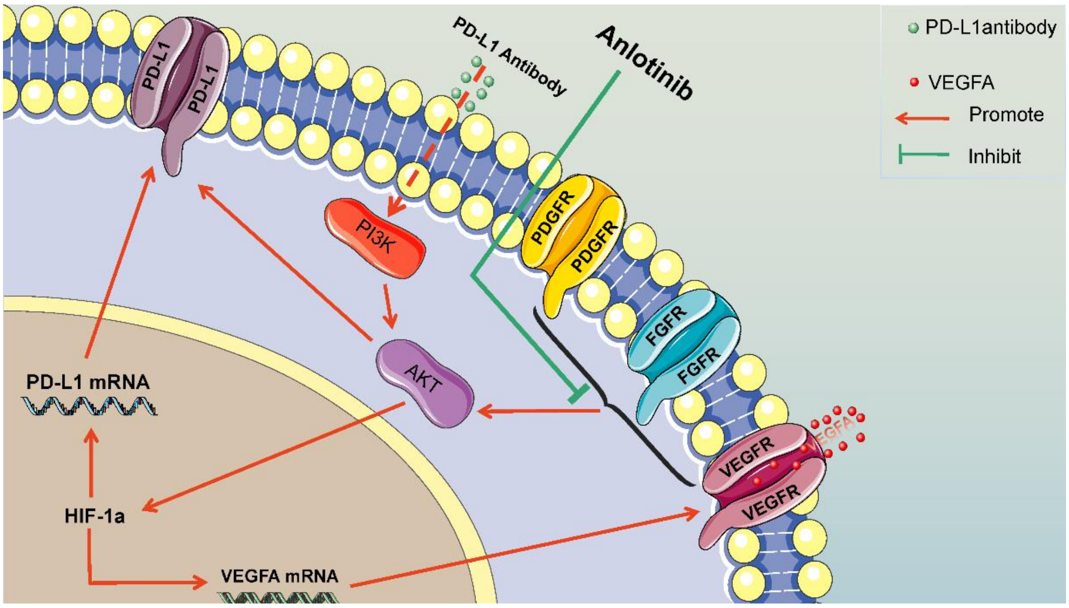

Supplement: Supplementary file 1 — Figure S1‐S8 [file CAM4-12-15337-s001.pdf]
