# Supplementary material for: Immune‐independent acquired resistance to PD‐L1 antibody initiated by PD‐L1 upregulation via PI3K/AKT signaling can be reversed by anlotinib
Source: Cancer Med. 2023 Jun 23;12(14):15337–49. doi: 10.1002/cam4.6195 (PMC10417303; doi:10.1002/cam4.6195)
Supplement: Supplementary file 2 — Table S1 [file CAM4-12-15337-s002.pdf]

List of qPCR primers sequence

mouse primers:

Primer name	Sequence (5’-3’)
PD-L1	F:GCTCCAAAGGACTTGTACGTG
	R:TGATCTGAAGGGCAGCATTTC
BCL-2	F: GCTACCGTCGTGACTTCGC
	R:CCCCACCGAACTCAAAGAAGG
VEGFA	F:CTGCTGTAACGATGAAGCCCTG
	R:GCTGTAGGAAGCTCATCTCTCC
HIF-1a	F:GGGGAGGACGATGAACATCAA
	R:GGGTGGTTTCTTGTACCCACA
BAX	F:AGGATGCGTCCACCAAGAAGCT
	R:TCCGTGTCCACGTCAGCAATCA
GAPDH	F:AGGTCGGTGTGAACGGATTTG
	R:GGGGTCGTTGATGGCAACA

human primers:

Primer name	Sequence (5’-3’)
PD-L1	F:TGCCGACTACAAGCGAATTACTG
	R:CTGCTTGTCCAGATGACTTCGG
BCL-2	F:ATCGCCCTGTGGATGACTGAGT
	R:GCCAGGAGAAATCAAACAGAGGC
VEGFA	F:TTGCCTTGCTGCTCTACCTCCA
	R:GATGGCAGTAGCTGCGCTGATA
HIF-1a	F:TATGAGCCAGAAGAACTTTTAGGC
	R:CACCTCTTTTGGCAAGCATCCTG
BAX	F:TCAGGATGCGTCCACCAAGAAG
	R:TGTGTCCACGGCGGCAATCATC
GAPDH	F:GTCTCCTCTGACTTCAACAGCG
	R:ACCACCCTGTTGCTGTAGCCAA
